# Selective Internal Radiotherapy Changes the Immune Profiles of Extracellular Vesicles and Their Immune Origin in Patients with Inoperable Cholangiocarcinoma

**DOI:** 10.3390/cells11152309

**Published:** 2022-07-27

**Authors:** Florian Haag, Anjana Manikkam, Daniel Kraft, Caroline Bär, Vanessa Wilke, Aleksander J. Nowak, Jessica Bertrand, Jazan Omari, Maciej Pech, Severin Gylstorff, Borna Relja

**Affiliations:** 1 Experimental Radiology, Department of Radiology and Nuclear Medicine, Otto-von-Guericke University Magdeburg, 39120 Magdeburg, Germany; florian.haag@med.ovgu.de (F.H.); anjana.manikkam@st.ovgu.de (A.M.); daniel.kraft@st.ovgu.de (D.K.); caroline.baer@med.ovgu.de (C.B.); vanessa.wilke@st.ovgu.de (V.W.); aleksander.nowak@med.ovgu.de (A.J.N.); jazan.omari@med.ovgu.de (J.O.); maciej.pech@med.ovgu.de (M.P.); severin.gylstorff@med.ovgu.de (S.G.); 2 Research Campus STIMULATE, Otto-von-Guericke University, 39106 Magdeburg, Germany; 3 Department of Orthopaedic Surgery, Otto-von-Guericke University, 39120 Magdeburg, Germany; jessica.bertrand@med.ovgu.de

**Keywords:** CCA, EVs, biomarker, MHC, diagnosis, prognosis

## Abstract

The incidence of cholangiocellular carcinoma (CCA) is rising worldwide. As there are no specific early symptoms or specific markers of CCA, it is often diagnosed in later inoperable stages. Accumulating evidence underlines the importance of radiation therapy in the induction of antitumor immunity. The surface protein composition on extracellular vesicles (EVs) relates to originating cells and thus may play a role in vesicle function. We assessed immune profiles of EVs and their immune origin in patients with inoperable CCA prior and after selective internal radiotherapy (SIRT). A total of 47 CCA patients receiving SIRT and 12 healthy volunteers (HV) were included. Blood was withdrawn before therapy (pre T) and after T. EVs were purified from plasma by cluster of differentiation (CD)9-, CD63-, and CD81-immunobead isolation. To detect differently abundant surface markers, dynamic range and EVs input quality were assessed. A total of 37 EVs surface markers were measured by flow cytometry and correlated either with the administered activity dose (MBq) or with the interval until death (month). EVs phenotyping identified lymphocytes, B cells, NK cells, platelets, endothelial cells, leukocyte activation, B cell activation, T and B cell adhesion markers, stem/progenitor cells, and antigen-presenting cells (APC) as EVs-parenteral cells. CD4 and CD8 significantly declined, while other markers significantly increased in CCA patients pre T vs. HV. Platelets-deriving EVs significantly decreased, normalizing to levels of HV but still significantly increasing vs. HV post SIRT. B cells-deriving EVs significantly increased pre T vs. HV, positively correlating with administered activity dose. MHCII and CD40 EVs significantly increased pre SIRT and negatively correlated with administered activity dose, while EVs from antigen presenting cells and CD49e pre SIRT positively correlated with survival time after therapy. Increased levels of CD24 and CD44 in cancer pre T were significantly decreased post T. Among the heterogeneity of EVs that was demonstrated, in particular, B cells-deriving, MHCII, and CD40 positive or APC-deriving EVs need to be further studied for their diagnostic or prognostic relevance in clinical scenarios.

## 1. Introduction

Cholangiocellular carcinoma (CCA) is a heterogenous group of epithelial malignancies of the intra- and extrahepatic bile ducts or the bile gland. Since its classification is based on the anatomic location with regards to the liver, three subtypes can be stratified: intrahepatic, perihilar (Klatskin tumor), and distal extrahepatic [1]. CCA has a rising incidence worldwide which is mainly caused by its risk factors such as liver cirrhosis and obesity [1]. Intrahepatic CCA is the second most common primary liver cancer after hepatocellular carcinoma [1]. The five-year survival rate ranges between 7 and 20%, which estimates the outcome rather poorly [2]. Since symptoms mostly appear late, and due to the lack of reliable biomarkers, CCA is often diagnosed in the late cancer phase. Although carbohydrate antigen 19-9 and carcinoembryonic antigen is a common routine practice towards the diagnosis, they have low sensitivity and specificity with respect to detecting CCA in early stages [3]. On the other hand, the prognosis is very poor, as a late diagnosis bears a limited potential and extent of surgical resection and only a palliative therapy setting is possible [4,5]. Apart from the combination of cisplatin and gemcitabine, there is no “gold standard” chemotherapy regimen with strong evidence [6,7]. Therefore, there is an urgent need for novel diagnostic and therapeutic approaches towards the early detection and beneficial management of CCA.

An effective therapy approach that can also be applied in later stages of the disease, beside the use of checkpoint-inhibitors, is the selective internal radiation therapy (SIRT) which is a minimal invasive radio ablative therapy [8,9]. In SIRT, radioactive material (e.g., microspheres loaded with the radioisotope Yttrium-90) is delivered directly into the tumor’s bloodstream via an arterial catheter. The radiation leads to necrosis of the tumor tissue and invasion of immune cells [10]. It still remains controversial if, due to the immune response to a therapy, an abscopal effect (which describes immune cells getting primed by undergoing tumor cells, subsequently “attacking” out-of-the-field metastasis) can be observed [11]. This highlights the unique role of the immune system and intercellular communication in cancer diagnosis, therapy, and especially in ablative therapy procedures.

Extracellular vesicles (EVs), important mediators of cell-cell-communication, are microparticles with a size from 30 to 10,000 nm enclosed by a lipid bilayer that can be released by almost all types of cells, including cancer cells [12]. EVs contain intracellular products of their origin cells such as microRNA, messenger RNA (mRNA), deoxyribonucleic acids (DNA), and proteins, which are also functional in the recipient cells [13]. They have recently emerged as promising biomarkers and therapeutic targets in cancer research [14]. EVs can be classified into three main categories: exosomes, microvesicles, and apoptotic bodies [12]. The distinct type of EVs can be identified and isolated based of their surface antigens. Accordingly, cluster of differentiation proteins (CD)9, CD63, and CD81 expressed on exosomes are frequently applied for their isolation and characterization [15,16,17]. There is emerging evidence that circulating EVs play an important role in carcinogenesis and tumor microenvironment [18]. It was shown that different expression of proteins, lipids, or nucleic acids in EVs from cancer patients compared to healthy subjects often mirrors the type of cancer [19]. Webber et al. demonstrated that exosomes produced by cancer cells could transmit information to normal stromal fibroblasts and trigger a cellular response. Their work showed that transforming growth factor beta (TGFβ)1 expressed at the exosome surface induced differentiation from fibroblasts to tumor-promoting myofibroblasts which act pro-angiogenetic [20,21]. Furthermore, it was shown that the levels of circulating exosomes had a promising diagnostic potential in the context of pancreatic, hepatocellular, and ovarian carcinoma [22,23]. Also, the count of circulating exosomes has been demonstrated as a prognostic marker for lung cancer patients in a meta-analysis [24]. The expression of surface antigens, notably CD105 and CD146 on EVs had a significant prognostic potential in metastatic colorectal cancer [25]. There is evidence that EVs also play a role in origin and progression of CCA. Therefore, it can also be considered that their levels in the circulation of patients and surface proteins can also be useful as diagnostic and prognostic markers [26].

Hence, there is an urgent need for the discovery of novel diagnostic and prognostic biomarkers and therapeutic targets towards the fast detection and management of CCA therapeutic regimes. Here, we performed a multiplexed phenotyping of EVs determining their immune profiles and their immune origin in patients with inoperable CCA prior and after SIRT with the aim to identify new molecular players that could be used as biomarkers and eventually therapeutic targets in future. EVs were purified from plasma by CD9−, CD63−, and CD81−immunobead isolation, and the results demonstrate that multiple cell types associated with the circulatory system contribute to the heterogeneity of EVs before and after therapy. Moreover, they may be applied as diagnostic and/or prognostic markers.

## 2. Materials and Methods

### 2.1. Ethics

This study was performed in the University Hospital of University Magdeburg with institutional ethics committee approval (SWARM RAD298), in accordance with the Declaration of Helsinki. All patients included gave written informed consent.

### 2.2. Study Settings and Population

Forty-seven patients (male n = 27, female n = 20, median age 71.00 (60.50–78.50) years undergoing selective internal radiotherapy of CCA. Forty-four patients had intrahepatic CCA. One patient had extrahepatic CCA. Two patients had CCA combined with another tumor entity (prostate carcinoma, hepatocellular carcinoma). Inclusion criteria were: (i) patient with CCA, (ii) indication for radioembolization (RE)/SIRT, (iii) chemotherapy and cortisone therapy paused for minimum two week before admission, (iv) >18 years of age. Exclusion criteria were (i) live expectancy < 3 months, (ii) hepatic tumor load > 70%, (iii) chronic infections, (iv) pronounced ascites, (v) contraindications for angiography, MRI contrast medium (Gd-EOB-DTPA), X-ray contrast medium, MRI and CT, (vi) severe cardiovascular diseases (NYHA III/IV), (vii) thrombotic or embolic events in the last 6 months (stroke/TIA), (viii) immunosuppression (e.g., in Z.n. transplantation) or HIV, especially cortisone long-term therapy, and (ix) autoimmune diseases or chronic inflammatory bowel diseases.

### 2.3. Technique of 90Y-Radioembolization

A detailed description of RE/ SIRT was previously provided [27]. RE was performed using Yttrium-90 (90Y) resin microspheres (SIR-Spheres^®^, Sirtex Medical, Lane Cove, Australia). Before RE the hepatic arterial tree and the arterial feeders to the gastrointestinal tract were identified via angiography. The gastroduodenal and right gastric arteries as any other gastrointestinal tract feeders were embolized via coils or plugs to isolate the hepatic arterial blood supply. In the next step 99mTc-MAA (150 MBq, 99mTc-LyoMAA, Covidien, Neustadt/Donau, Germany) was delivered into the left and right hepatic artery and a gamma camera (E.CAM 180, Siemens, Erlangen, Germany) determined the extent of hepatopulmonary shunting and tumor covering. A SPECT scan of the upper abdomen was performed to identify nontarget extrahepatic seeding of 99mTc-MAA. Activity of 90Y resin microspheres was calculated by the body surface area (BSA) method. Up to 2 weeks later, 90Y resin microspheres were delivered selectively into the hepatic arteries via a temporary transfemoral catheter placed selective in the left and right hepatic artery.

### 2.4. Data Acquisition and Blood Sampling

**Figure 1:**
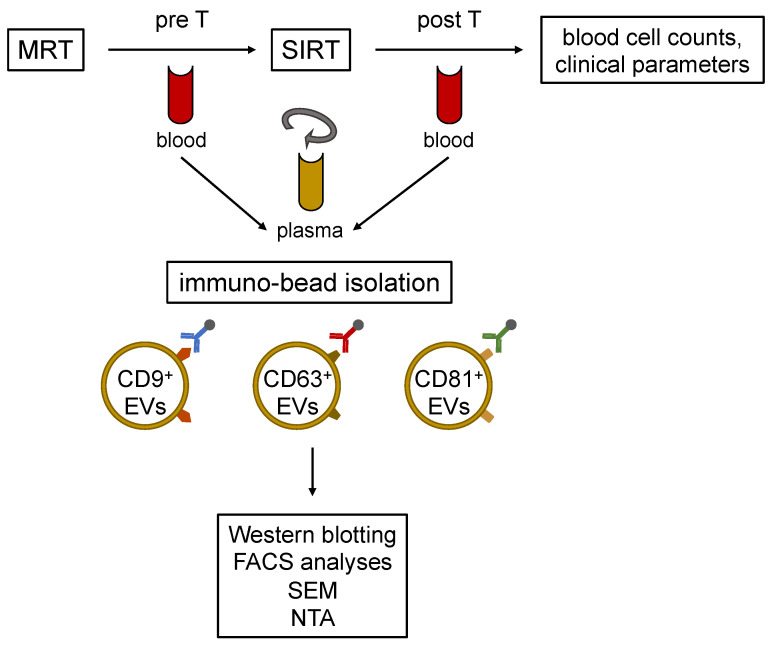
Schematic illustration of the experimental workflow. Cholangiocellular carcinoma (CCA) patients received a liver MRI according to the clinical standards. Blood samples were taken before (pre T) and after (post T) selective internal radiation therapy (SIRT). Extracellular vesicles (EVs) were isolated from plasma using CD9+, CD63+, and CD81+ exosome markers in the immune-bead isolation. The isolated EVs populations are distinguishable by flow cytometry (FACS). The presence of EVs was assessed by western blotting, and in addition to the characterization of EVs, their morphology was analyzed by scanning electron microscopy (SEM) and nanoparticle tracking analysis (NTA).

The laboratory evaluation one day prior to SIRT and two days after SIRT was performed. Blood samples were collected in ethylenediaminetetraacetic acid (EDTA) tubes (Becton Vacutainer, Becton Dickinson Diagnostics, Aalst, Belgium) and citrate tubes (Becton Vacutainer) before (pre T) and after (post T) SIRT. After blood drawing, EDTA-blood was kept vertically on room temperature avoiding agitation. In the case of multiple SIRTs, plasma was also drawn before and after the following therapies. Blood was centrifuged at 2000× *g* for 15 min at 4 °C and plasma was stored at −80 °C until the EVs isolation and sample analysis (Figure 1). Twelve healthy volunteers served as controls. Blood counts (leukocytes, erythrocytes, red cell distribution width, thrombocytes, neutrophil granulocytes, immature granulocytes, eosinophil granulocytes, basophil granulocytes, lymphocytes and monocytes) were determined in the university hospital clinical routine as well as the coagulation parameters (quick value, international normalized ratio (INR), partial thromboplastin time (PTT) and thrombin time), clinical chemistry (creatinine, urea, uric acid, bilirubin, albumin, alanine aminotransferase, aspartate aminotransferase, alkaline phosphatase and gamma-glutamyl transferase) and c-reactive protein (CRP) pre SIRT. Blood was collected in serum tubes (clinical chemistry and CRP) and EDTA tubes (blood count).

### 2.5. EV Isolation

EV isolation was performed using magnetic-activated cell sorting (MACS). For the isolation one ml plasma sample was pre-cleared by dilution in 1 mL phosphate buffered saline (PBS) buffer (Thermo Fisher Scientific, Waltham, MA, USA) and repeated centrifugation was performed (2000× *g* for 30 min and 10,000× *g* for 45 min at room temperature). The pre-cleared samples w ere incubated with 50 µL magnetic labelled beads (Exosome Isolation Kit Pan, human, #130-110-912, Miltenyi Biotec, Bergisch Gladbach, Germany) and incubated for one hour at room temperature. After incubation the samples were loaded into magnetic columns (µ Columns with plungers, #130-110-905, Miltenyi Biotec), which were placed in a thermoMACS separator (#130-091-136, Miltenyi Biotec), equilibrated with 100 µL of equilibration buffer (Exosome Isolation Kit Pan, human, #130-110-912, Miltenyi Biotec) and rinsed three times with isolation buffer (Exosome Isolation Kit Pan, human, #130-110-912, Miltenyi Biotec). After the sample run through the columns, the columns were washed four times with 200 µL of isolation buffer. This way the magnetic labelled EVs sticked to the columns and were separated from the waste. After the samples and the isolation buffer completely passed the columns, they were placed in 1.5 mL tubes, 100 µL of isolation buffer were added and the EVs were flushed out in the tubes. After isolation the samples were stored at −80 °C.

### 2.6. EV Measurement via FACS

The isolated EVs were incubated with MACSPlex Exosome capture beads, deriving from MACSPlex Exosome kit (#130-108-813, Miltenyi Biotec) and incubated overnight on an orbital shaker (450 rpm, room temperature). At the next day one ml of MACSPlex buffer was added (MACSPlex Exosome kit, #130-108-813, Miltenyi Biotec) and the sample was centrifuged (3000× *g*, 5 min, room temperature). One ml of the supernatant was discarded and 135 µL were left in the tube. To the leftover 15 µL of MACSPlex Exosome detection reagent mastermix, consisting of 5 µL detection reagent for CD9, 5 µL detection reagent for CD63 and 5 µL detection reagent for CD81 (MACSPlex Exosome kit, #130-108-813, Miltenyi Biotec), were added. The sample was incubated at room temperature for one hour and after that centrifuged at 3000× *g* and room temperature for 5 min. Following the centrifugation one ml of supernatant was discarded and one ml of MACSPlex buffer was added. The following 15 min the sample was incubated in the dark on an orbital shaker (450 rpm). The incubated sample was centrifuged at 3000× *g* for 5 min at room temperature. One ml of supernatant was discarded. The leftover was and studied via FACS. Data assessed by flow cytometry were normalized to control markers provided in the assay as suggested by the manufacturer, and APC median signal intensities between the groups were compared. Western blot results were normalized to baseline TSG101 expression and then referred to HV.

### 2.7. Western Blotting

The total protein concentration of MACS-isolated EVs was quantified using Lowry Protein Assay [28]. 10 µg of the EV fractions were lysed directly by sodium dodecyl sulfate (SDS) sample buffer (200 mM Tris-HCL (pH 6.8); 10% SDS; 0.4% bromophenol blue; 40% glycerol) and was separated by SDS (12%) gel electrophoresis (SDS-PAGE). For the detection of CD9 and TSG101 signal the EV fraction proteins were transferred onto 0.45 µm PVDF-membranes (Cytiva, 10600023, Marlborough, MA, USA). Membranes were blocked with 5% milk powder and 0.1% Tween in Tris-Buffered Saline (TBS) for 1 h at room, followed by primary antibody incubation overnight at 4 °C. The horseradish peroxidase (HRP)-coupled secondary antibodies were incubated for 1 h at room temperature. Subsequently, enhanced chemiluminescence (Millipore WBKLS0500, Merck KGaA, Burlington, MA, USA) detection and visualization were implemented and detected by Octoplus QPLEX imager from NH DyeAgnostics (Halle, Germany). For semi-quantitative analysis of western blot signals, ImageJ software was used. The following antibodies were applied: CD9 (1:1000 Purified mouse monoclonal anti-human, Mouse IgG1, κ, 312102 Biolegend, San Diego, CA, USA), TSG101 (1:200 tsg 101 mouse monoclonal antibody, sc-136111, Santa Cruz, Dallas, USA), goat-anti-mouse-HRP (1:1000 dilution, polyclonal, ab205719, Abcam, Cambridge, UK).

### 2.8. Dynamic Light Scattering

The Size of the EVs was assessed by Zetasizer Nano (Zetasizer Nano, ZEN 3600 Malvern Instruments Ltd., Malvern, UK). The extracted EV fractions were diluted 1:100 using sterile filtered PBS and measured in polystyrene cuvette (67.745, Sarstedt AG & Co. KG, Nümbrecht, Germany). For reproducibility and standardization of the EV size, the parameter values were used as follows; a material refraction index of blood EVs of 1.39 and a dispersant refraction index of water of 1.330 [29]. The samples were incubated for 180 s and measured while temperature was controlled at 25 °C. The Zetasizer Nano used a laser wavelength of 632.8 nm at a 90° fixed angle. The size or homogeneity of the EVs was recognized through the diameter and the intensity in per cent, respectively. Each measurement with DLS was conducted with 10 acquisitions of 5 s; the diameter was measured between 1 and 10,000 nm. The measurement settings were adjusted and tested with carboxylated polystyrene calibration particles (Calibration Particles—qNano, Izon Science Ltd., Lyon, France) between 70 and 400 nm. N represents the number of EV samples from each patient sample, and n represents the number of collected data (HV: n = 3, n = 9, TP: n = 3, n = 9).

### 2.9. Scanning Electron Microscopy Analysis

In preparation for SEM, coverslips (Thermo Fischer Scientific Inc.) were cleaned with acetone, ethanol, and ultrapure water and then coated with 100 µg/mL poly-D-lysine (Millipore A003E, Merck KgaA) solution for 24 h at 4 °C [30]. The EV samples were adhered to poly-D-lysine coated glass coverslips and incubated overnight [31]. After washing with DPBS the EVs were then fixed with 4% formaldehyde and incubated for 2 h at room temperature. After washing with PBS and water followed by gradual dehydration from 70% to 100% ethanol in water with a 10% concentration increment step every 5 min [30]. The EV samples were then coated with gold (<10 mm) to increase the image contrast, enhance the electric conductivity, and avoid surface charging. The coated coverslips were mounted (sample facing upwards) on the microscope slide. The images were captured by the Scanning Electron Microscope (FEI Scios DualBeam equipped with an EDAX EDS system, Thermo Fischer Scientific Inc.) at 10–12 KeV voltages 10,000× and 35,000× resolution.

### 2.10. Statistics

GraphPad Prism 6.0 software (GraphPad Software Inc. San Diego, CA, USA) was used to perform the statistical analysis. The normality of all data was verified by the Kolmogorov-Smirnov test. Data are given as mean ± standard error of the mean or as otherwise indicated. The differences between the healthy volunteers versus tumor patients were determined by the non-parametric Mann-Whitney test. The statistically significant differences between the EVs in patients before versus after therapy were assessed by the nonparametric Wilcoxon matched-pairs signed rank test. The correlation analyses were performed by determining the Spearman correlation significance and Spearman r. A *p* value below 0.05 was considered statistically significant.

## 3. Results

### 3.1. Data Description

#### 3.1.1. Patient Cohort

A total of 47 Patients (20 female, 27 male) with a median age of 71 years were included. Total liver volume (median: 1888 cm^3^), tumor volume (median: 379.5 cm^3^), tumor fraction (median: 21.19%), and administrated activity dose (median: 1055 MBq) were raised. The median values of albumin, alanine aminotransferase, aspartate aminotransferase, quick valve, INR, PTT, thrombin time were within the standard range. Median values of alkaline phosphatase, gamma-glutamyl transferase, and CRP were increased (Table 1).

#### 3.1.2. Immune Cell Status

The included patients showed a tendency to increased leukocyte numbers and neutrophil granulocytes after therapy. Counts of erythrocytes and thrombocytes were significantly decreased after SIRT vs. pre T (*p* < 0.05, Table 2). The percentage of lymphocytes and monocytes decreased post T vs. pre T, however this was not significant.

#### 3.1.3. Verification of Isolated EVs

**Figure 2:**
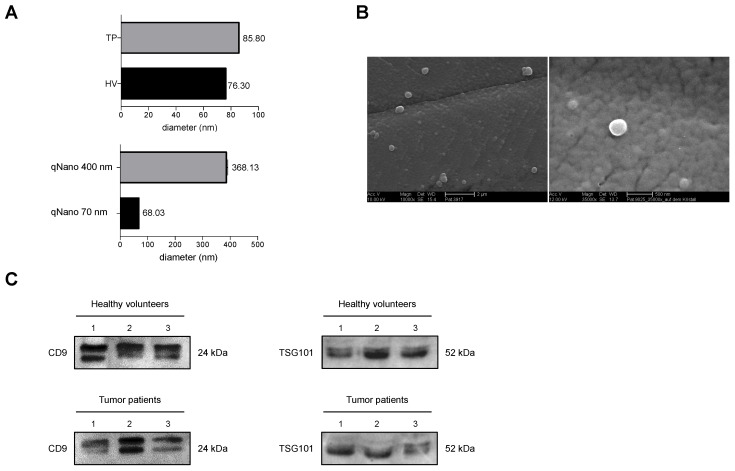
Assessment of the quality of isolated extracellular vesicles (EVs) from healthy volunteers (HV) and tumor patients (TP) one day before (pre T) and two days (post T) after selective internal radiotherapy radioembolization. (**A**) Representative DLS- size distribution profile of calibration nanoparticles and isolated EVs collected in HV and TP. (**B**) Scanning electron microscopy image of of isolated EVs. The scale bar represents 2 µm, (10,000×; AccV. 10 kV) and 500 nm (35,000×; AccV. 12 kV) respectively. (**C**) Representative western blot assessing the protein content of isolated EVs from plasma of three different HV or TP (1–3) using genuine EVs markers CD9 and TSG101.

The average diameter of isolated EVs measured with DLS showed EVs in the expected range of 30–250 nm of small EVs and exosomes. It was revealed that the average diameter of EVs from HV was 76.3 nm. In comparison, those from the TP was 85.8 nm (Figure 2A). Furthermore, the morphology and size of exosomes were verified by scanning electron microscopy (Figure 2B). The results confirmed the sphere-shaped vesicles, the same as the known morphology of exosomes and small EVs. CD81, CD9 and CD63 were found in samples from HV and TP after Macs Isolation (Figure 1). There were no significant changes in expression levels of CD9-, CD81-, or CD63-positive EVs among HV and TP (data not shown). Similarly, the Western blot protein analysis of EVs samples showed a positive CD9 signal as well as a positive TSG101 signal as internal controls (Figure 2C).

#### 3.1.4. Immune Origin of EVs

**Figure 3:**
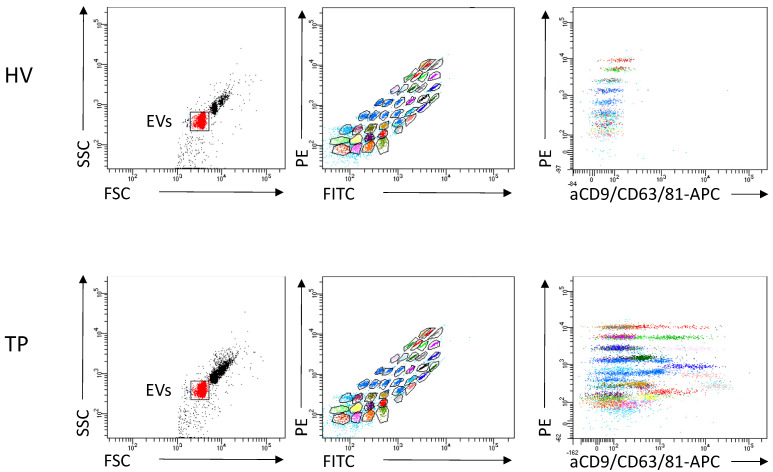
Representative flow cytometric gating strategy for extracellular vesicles (EVs) is shown for healthy volunteers and tumor patients (TP). Analysis examples show exclusion of doublets and no bead events, (**left**) discrimination of differently labelled bead populations (**middle**), and measurement of signal intensities of the single bead populations (**right**). The positive bead populations are highlighted in colours.

The immune origin of EVs was assessed as representatively shown in Figure 3.

**Figure 4:**
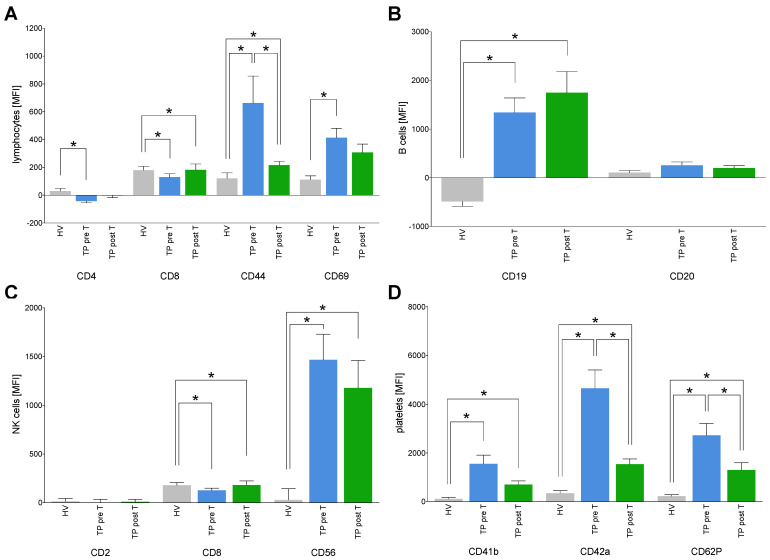
Expression of cluster of differentiation (CD) surface proteins on isolated extracellular vesicles (EVs) specific for lymphocytes (**A**), B cells (**B**), NK cells (**C**) and platelets (**D**). The EVs derived from healthy volunteers (HV) and cholangiocellular carcinoma (CCA) tumor patients (TP) before (pre T) and after (post T) receiving a selective internal radiation therapy. The mean fluorescent intensity (MFI) is given as mean ± standard error of the mean. * *p* < 0.05 vs. indicated group.

CD4, CD8, CD44, and CD69 are typically expressed on the surface of lymphocytes [32]. CD4 and CD8 presence on EVs was significantly reduced in TP pre T vs. HV (*p* < 0.05, Figure 4A). CD8 was significantly increased in TP post T vs. HV (*p* < 0.05, Figure 4A). CD44 was significantly enhanced in TP both pre T as well as post T vs. HV (*p* < 0.05, Figure 4A). However, CD44 was significantly reduced post T vs. pre T in TP (*p* < 0.05, Figure 4A). CD69 was significantly increased in TP pre T vs. HV (*p* < 0.05), while there was no significant difference in TP post T vs. HV (Figure 4A).

CD19 is typically expressed on the surface of B cells [33]. CD19 was significantly enhanced in TP both pre T as well as post T vs. HV (*p* < 0.05, Figure 4B).

As marker of natural killer (NK) cells [33], CD56 was significantly enhanced in TP both pre T as well as post T vs. HV (*p* < 0.05, Figure 4C).

CD41b, CD42a, and CD62P are expressed on the surface of platelets [33]. All three markers were significantly increased in TP pre T as well as post T vs. HV, respectively (*p* < 0.05, Figure 4D). After SIRT, CD42a and CD62P were significantly reduced in TP vs. pre T (*p* < 0.05, Figure 4D).

#### 3.1.5. Immune Activation Markers on EVs

**Figure 5:**
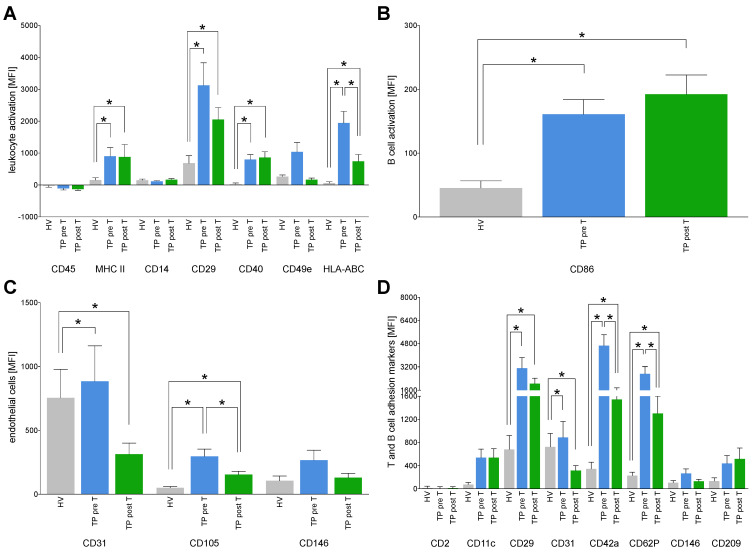
Expression of cluster of differentiation (CD) surface proteins on isolated extracellular vesicles (EVs) specific for leukocyte activation (**A**), B cells (**B**), endothelial cells (**C**), and T and B cell adhesion markers (**D**). The EVs derived from healthy volunteers (HV) and cholangiocellular carcinoma (CCA) tumor patients (TP) before (pre T) and after (post T) receiving a selective internal radiation therapy. The mean fluorescent intensity (MFI) is given as mean ± standard error of the mean. * *p* < 0.05 vs. indicated group.

Expression of several specific antigens as, e.g., MHC II, CD14, CD29, CD40, CD49e, or HLA-ABC is associated with leukocyte activation (22,24–26). CCA patients showed significantly increased levels of MHC II, CD14, CD29, CD40, and HLA-ABC on EVs in TP both pre T and post T compared to HV (*p* < 0.05, Figure 5A). HLA-ABC expression on EVs was significantly reduced in TP post T vs. TP pre T (*p* > 0.05, Figure 5A).

CD86 as a typical marker of activated B-cells [33] is significantly increased in TP both pre T as well as post T vs. HV, respectively (*p* > 0.05, Figure 5B).

As CD31, CD105 and CD146 are typically expressed on endothelial cells [32], they were clustered to detect potential EVs originating from endothelia. CD31 and CD105 were significantly increased in TP pre T vs. HV, while the SIRT was associated with significantly reduced expression levels of CD31 and CD105 on EVs in TP post T vs. TP pre T and vs. HV, respectively (*p* < 0.05, Figure 5C).

Further markers that are involved in processes of T and B cell activation and adhesion to endothelia such as CD2, CD11c, CD29, or CD209 were analyzed [22]. CD29, CD31, CD42a, CD62P were significantly increased in TP pre T as well as post T vs. HV (*p* > 0.05, Figure 5D). Other measured markers on EVs have shown a tendency to be increasingly expressed on EVs. However, those changes were not significant. CD42a and CD62P were significantly decreased in TP post T vs. pre T (*p* < 0.05, Figure 5D).

#### 3.1.6. Expression of Tumor Markers on EVs

**Figure 6:**
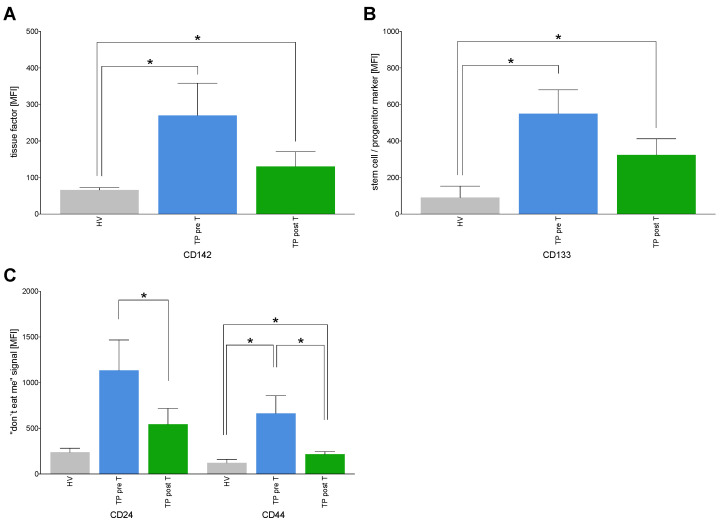
Expression of cluster of differentiation (CD) surface proteins on isolated extracellular vesicles (EVs) specific for tissue factor (**A**), stem cells/progenitor marker (**B**), and “don’t eat me signals” (**C**). The EVs derived from healthy volunteers (HV) and cholangiocellular carcinoma (CCA) tumor patients (TP) before (pre T) and after (post T) receiving a selective internal radiation therapy. The mean fluorescent intensity (MFI) is given as mean ± standard error of the mean. * *p* < 0.05 vs. indicated group.

CD142, also known as tissue factor (TF) plays a role in hemostasis and inflammation [34]. Its expression was significantly increased on EVs deriving from TP pre T as well as post T compared to HV (*p* < 0.05, Figure 6A). SIRT reduced CD142 expression on EVs, however, this change was not significant (Figure 6A).

CD133 is a marker for stem cells and progenitor cells [35]. Comparable to CD142, CD133 expression on EVs was significantly higher in TP both pre T as well as post T vs. HV (*p* < 0.05, Figure 6B).

CD24 and CD44 expression has been described in tumors before, and they are involved in tumor survival and growth [36]. While CD24 showed a tendency to a higher expression in EVs in TP pre T as well as post T vs. HV, CD44 expression on EVs was significantly increased in TP pre T as well as post T vs. HV (*p* < 0.05, Figure 6C). SIRT reduced CD24 as well as CD44 presence on EVs in TP post T as compared the samples originating from TP pre T (*p* < 0.05, Figure 6C).

#### 3.1.7. Correlation Analyses of the Expression Markers on EVs with the Administered Activity Dose as Well as Interval until Death

Correlation analyses has shown significant positive correlations in the expression levels of B cell markers CD19 and CD20 on EVs deriving from TP pre T with the administered activity dose (MBq) (CD19: Spearman r = 0.3776, *p* < 0.05; CD20: Spearman r = 0.3790, *p* < 0.05, Table 3).

EVs expression levels of CD45 that is present in various isoforms on all differentiated hematopoietic cells have shown a significant negative correlation in TP post T with the administered activity dose (Spearman r = −0.4881, *p* < 0.05, Table 3). Expression of MHC II that is normally found on professional antigen-presenting cells has shown a significant negative correlation in TP pre T with the administered activity dose (Spearman r = −0.4718, *p* < 0.05, Table 3). CD29 expression on EVs in TP pre T has shown a significant positive correlation with the administered activity dose (Spearman r = 0.0181, *p* < 0.05, Table 3).

CD40 that is found on antigen-presenting cells and required for their activation showed a significant negative correlation in TP pre T with the administered activity dose (Spearman r = −0.3594, *p* < 0.05, Table 3).

CD11c typically expressed on leukocytes has shown a significant negative correlation in TP post T with the administered activity dose (Spearman r = −0.4952, *p* < 0.05, Table 3).

CD209 that is present on the surface of both macrophages and dendritic cells and involved in endothelial adhesion and activation of CD4+ T cells, has shown a significant positive correlation in TP pre T with the administered activity dose (Spearman r = 0.4003, *p* < 0.05, Table 3).

The TF CD142 that is also present in subendothelial tissue and leukocytes has shown a significant positive correlation in TP pre T with the administered activity dose (Spearman r = 0.3579, *p* < 0.05, Table 3).

MHC II on EVs originating from TP pre T has shown a significant positive correlation in TP pre T with the interval until death (Spearman r = 0.5483, *p* < 0.05, Table 3).

CD49e that is involved in adhesion processes and cell-surface mediated signaling has shown a significant positive correlation in TP pre T with the interval until death (Spearman r = 0.5506, *p* < 0.05, Table 3).

#### 3.1.8. Exemplary Confirmation of Flow Cytometry Data by Western Blot

**Figure 7:**
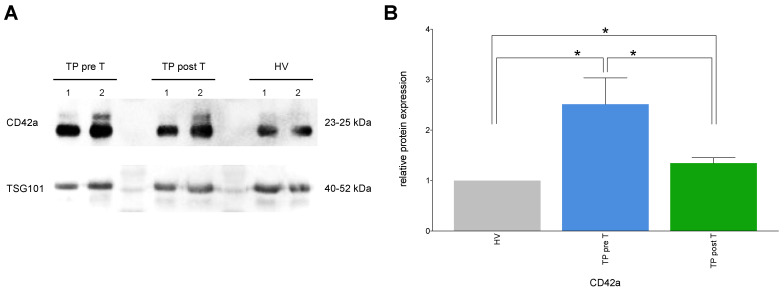
Representative protein expression of the cluster of differentiation (CD)42a and internal control TSG101 in western blot analysis (**A**), and relative changes in the protein expression levels of CD42a (**B**) in isolated EVs. The EVs derived from healthy volunteers (HV) and cholangiocellular carcinoma (CCA) tumor patients (TP) before (pre T) and after (post T) receiving a selective internal radiation therapy. The mean fluorescent intensity (MFI) is given as mean ± standard error of the mean. * *p* < 0.05 vs. indicated group.

CD42a and internal control for EVs TSG101 were analyzed by western blot to exemplary verify the results obtained by flow cytometric analyses (Figure 7). As shown in Figure 4D, also in western blot protein expression analysis (Figure 7A), and subsequent normalization to TSG101 in HV, CD42a was significantly increased in TP pre T as well as post T vs. HV, respectively (*p* < 0.05, Figure 7B). After SIRT, CD42a was significantly reduced in TP vs. pre T (*p* < 0.05, Figure 7B).

## 4. Discussion

CCA is a heterogenous group of epithelial malignancies of the intra- and extrahepatic bile ducts or the bile gland [4]. It shows a rising incidence worldwide and thus is becoming more and more clinically relevant [1]. The only curative approach to the treatment of CCA is liver resection. Due to the advanced stage, only about one third of CCAs are resectable at diagnosis [2]. Even if a resection of the liver can be performed, recurrence rates of up to 60% are seen [37]. Reasons for this include, for example, microscopic liver metastases [38]. An effective non-curative therapy approach, which can also be performed in the later stages and improve the overall survival is SIRT [8]. Against this clinical background, it is obvious that it is of enormous importance to improve the diagnosis of CCA and to enable early detection, just as it is important to improve therapeutic approaches and strategies. A promising approach to this is the determination of EVs in the circulation of CCA patients. As they play an important role in tumor microenvironment and are, for example, important for angiogenesis [18,21,39], several studies were able to reported their diagnostic and prognostic potential [22,24,25].

Thus, our study provides novel insights regarding the characterization and origin of EVs in patients with inoperable CCA prior and after SIRT. Furthermore, their correlations with clinical parameters were identified. To enable an easy and fast screening of multiple surface proteins on EVs, different capture antibody beads were combined to assess the markers by flow cytometry. In addition to the characterization of EVs, we aimed to visualize the morphology of EVs by scanning electron microscopy. Furthermore, nanoparticle tracking analysis allowed us to determine the size of the isolated and analyzed EVs. The isolated EVs have shown a size distribution of at least 100 nm. For safe EVs isolation, CD9, CD63, and CD81, which are typically highly expressed on exosomes were applied [12]. We found that multiple cell types associated with the circulatory system contribute to the heterogeneity of EVs in CCA, and moreover may be potentially applied as diagnostic as well as prognostic markers (something which needs to be further elaborated in larger studies). The detected EVs profile indicated that different subsets of leukocytes including T cells and antigen presenting cells but also endothelial cells and platelets release EVs in CCA patients, and that this release is modified after SIRT.

Since it has been shown that EVs are involved in multisystemic signaling mediating regeneration and long-term adaptive responses [40,41], based on our findings as well, it is reasonable to assume that SIRT of the liver could have systemic implications on the immune system. In a recent study, a decrease in CD4 and CD8 carrying EVs was demonstrated, while the number of CD69 carrying EVs in the circulation of CCA patients was increased compared to HVs. CD4 and CD8 are typical markers for T cells and CD69 is a activation marker for T cells [42]. Gerwing et al. were also able to show a decrease of T helper cell derived EVs (CD4 positive) and a parallel increase of cytotoxic T cell derived EVs in an in vivo tumor model [43]. This underlines the hypothesis that the distribution of EVs in cancer might follow a specific pattern that may be suitable for diagnostic and prognostic applications. In line with this, Oba et al. suggested T cell derived EVs as a marker for the activity of several T cell subsets [44]. Specifically, in CCA patients raised levels of circulating CD19 and CD20 expressing EVs as typical B cell markers [45] were demonstrated. In line with this report, our data also indicate that CCA patients show increased levels of B cell derived EVs in circulation. Interestingly, our data also indicate an increased activation of B cells in CCA patients, since CD86 a marker for the activity of B cells and monocytes [46,47] was enhanced. SIRT did not significantly change the CD19 and CD86 presence on circulating EVs, but the observed tendency to an increase as well as the significant positive correlation of CD19 and CD20 with the clinically administered activity dose clearly indicate that B cell activation in SIRT might have relevant clinical impact and should be further elaborated in future studies. Specific patterns were also visible in the expression profiles of potentially NK cell derived EVs. CD2 is expressed on NK and T cells and plays an important role in their activation [48,49], while CD56 is a marker for their cytotoxicity [50]. CD56 expressing EVs in the circulation of CCA patients were enhanced compared to HVs, and not specifically changed by SIRT. There are ongoing approaches to apply NK cell stimulating substances in the therapy of CCA [51]. However, our current data do not reflect a significant relevance of SIRT as a potential stimulating factor of those cells or their enhanced communication via EVs in CCA patients. It is known that persistent antigen and inflammatory stimulation can cause T cell exhaustion, and thus that CD4 and CD8 T cells play a key role in the clearance of intracellular pathogens and tumors [52,53,54]. In this context they are part of the tumor infiltrating leukocytes in CCA [55]. An infiltration of CD8 T cells in the CCA is associated with higher survival and lower recurrence after surgery [56]. Also a greater level of circulating CD4 T cells is associated with a prolonged overall survival in patients with intrahepatic CCA undergoing radiation [57]. In our cohort of CCA patients, CD4 and CD8 expressing EVs were significantly reduced as compared with healthy volunteers, while SIRT reversed this effect. Since their expression levels did not correlate with the clinical outcome, the relevance of these SIRT induced changes remains unanswered. However, several other leukocyte activation markers that have been associated with the patient outcome [58,59,60] are significantly enhanced in CCA patients. Notably MHC II and HLA-ABC which are important for T cell activation [59,61] were strongly enhanced on circulating EVs from CCA patients. Furthermore, MHC II expression on EVs showed a negative correlation with the administered activity dose during SIRT as well as a positive correlation with the interval until death. SIRT strongly reduced the HLA-ABC presence on the EVs. It is known that HLA-ABC is crucial for proper presentation of specific antigens on the cancer cell surface for recognition by cytotoxic CD8 T cells [62], and that full activation of CD8 T cells requires both expression of HLA-ABC and expression of the T cell costimulatory molecule CD80 or CD86. In our study, CD8 expression on EVs was enhanced by SIRT, while HLA-ABC was reduced and CD86 remained enhanced, thus, whether SIRT has a positive or negative impact on the local expression of these factors directly in CCA cannot be answered and remains to be investigated. Comparable to the HLA-ABC expression, CD29 was also enhanced on circulating EVs from CCA patients and markedly reduced by SIRT. Considering that CD29 is also expressed on CCA tumor cell lines [63] und can be used as a cancer stem cell marker [64,65], it has to be further investigated whether the CD29 expressing EVs originate from activated leukocytes or tumor cells. The fact that CD29 prior SIRT positively correlated with the administered activity dose might support rather the local deliberation of CD29 loaded EVs and post SIRT observed changes in CCA patients. Beside leukocyte activation markers there were also changes in other endothelial as well as T and B cell adhesion markers, which were increased in CCA and significantly reduced by SIRT. As example, increased levels of circulating endothelial cell derived EVs expressing CD31, CD105 and CD146 [17,66] were detected in CCA patients. In line with our report, Brocco et al. described increased levels of CD31 positive EVs in non-hematological cancer patients [67]. Also it was shown that CD105 and CD146 positive EVs can play a role in the metastasis of breast cancer [68,69]. Interestingly, SIRT reduced CD105 expression on circulating EVs in CCA patients. In general, this study demonstrates a specific immune profile on circulating EVs from CCA patients including specific cell, activation and adhesion markers. However, identifying the specific origin of EVS as well as pre and post SIRT profiles of EVs in correlation with specific clinical outcome suggest an utmost clinical relevance of further studies.

Also, EVs expressing the platelet associated surface markers CD41b, CD42a, and CD62P [17,70] were increased in CCA patients and all together strongly reduced after SIRT. As platelets and platelet-derived messengers play an important role in the progression of CCA [71,72], our data indicate a beneficial impact of SIRT in CCA. However, a prognostic potential suspected in platelet derived EVs was not confirmed by this study. A possible positive effect of SIRT in CCA patients is supported by the assessment of CD24 and CD44 that can be expressed by tumor cells and play an important role in their adhesion, survival and growth [36]. It was shown before that increased CD24 expression on CCA cells is associated with tumor invasion, disease progression, lymph node metastasis and reduced overall survival. Therefore, CD24 expression on CCA cells has been suggested repeatedly as a prognostic marker for the outcome of CCA patients [73,74,75]. Another potential marker is CD44, which is associated with post-operative CCA recurrence in patients who were undergoing a surgical resection [76]. CD44 also plays a crucial role in proliferation, migration and invasion of the CCA [77]. Thus, our data showing enhanced expression of CD24 and CD44 on EVs from CCA patients is in line with these reports. Moreover, our data indicate a direct effect of SIRT in reducing CD24 and CD44 expression of EVs. Considering that tumor released EVs play a role in the prognosis and diagnosis of several tumor, our data indicate that SIRT induced EVs release in CCA might have a great importance. As CCA cells use EVs to interact with surrounding mesenchymal stem cells to modulate the microenvironment and enhance the tumor growth [78], it can be postulated that the reduction of these cell interactions via CD24 and CD44 loaded EVs after SIRT may reduce tumor growth and progression. Another finding supports a positive effect of SIRT in CCA patients. CD133, a surface marker expressed on stem cells and tumor progenitor cells [79], has been identified as a marker for tumor invasion, intrahepatic, and lymph node metastasis. Therefore, it is considered as a possible prognostic marker for worse outcome in CCA patients playing an important role in the communication of tumor cells with their surrounding [80,81]. Our data are in line with those reports, since CD133 expression on circulating EVs was significantly increased. The tendency to decreased CD133 expressing EVs after SIRT may indicate a reduced local communication of the tumor via EVs after SIRT. Similar expression pattern was observed for CD142 (TF) which is associated with intensification of inflammation and immunological processes during liver cirrhosis [34]. As Tseng et al. showed that CD142 expressing macrovesicles are associated with metastasis in lung cancer [82], the use of CD142 expressing EVs as an prognostic factor needs to be further investigated.

In the underlying study, the EVs surface markers were correlated with the administered activity dose (MBq) or with the interval until death. However, the application of EVs markers for possible diagnostic or predictive purposes in the clinics should imply correlations with additional clinical parameters in future and larger studies. As example, in the underlying study, we can provide the UICC (Union for International Cancer Control) stages, but unfortunately we do not have the complete data set of all included patients. Of the patients included, 0 were in stage I, 3 were in stage II, 4 were in stage IIIa, 3 were in stage IIIb and 23 were in stage IV at the start of therapy. The stages of the remaining patients are unknown. Also, the response to therapy should be considered in further studies, as of the patients included in the underlying study, 7 received chemotherapy after SIRT, 14 received a following SIRT, one received a TACE, 4 an ablation, 5 received none of these therapies, and the follow-up of the remaining cohort is unknown. Thus, these important issues should be considered in future cohorts to provide reliable evidence for clinical correlations.

## 5. Conclusions

In conclusion, we provide evidence that multiple cell types associated with the circulatory system contribute to the heterogeneity of EVs in CCA. This study suggests a complex EVs signaling network with implications in tumor antigen response, vascular functionality, immune modulation (including innate and adaptive immunity), platelet function and regeneration. Since heterogeneous changes are induced by SIRT, of which some are associated with improved outcomes in cancer, the severe impairment of cellular EVs in cancer signaling mechanisms and tissue crosstalk as well as mode of action in target cells regarding the clinical impact should be assessed in future studies. Identification of the underlying mechanisms associated with possible benefits of the applied clinical therapy approach such as SIRT may translate into future diagnostic and/or therapeutic applications of EVs.

## Figures and Tables

**Table 1:** Baseline characteristics and standard laboratory parameter. Median value with 75% percentile is given. Abbreviations: INR, international normalized ration; PTT, partial thromboplastin time.

Variables	n = 47
Age [median (range)]	71.00 (60.50–78.50)
Gender (female, n)	20
Total liver volume (cm^3^) [median (range)]	1888 (1434–2629)
Tumor volume (cm^3^) [median (range)]	379.50 (117.40–619.60)
Tumor fraction (%) [median (range)]	21.19 (9.09–26.62)
Administered activity dose (MBq) [median (range)]	1055.00 (871.30–1461.00)
Creatinine (umol/L) [median (range)]	69.00 (54.50–86.00)
Urea (mmol/L) [median (range)]	5.00 (3.90–6.50)
Uric acid (umol/L) [median (range)]	294.50 (239.30–335.80)
Bilirubin (umol/L) [median (range)]	6.85 (5.17–11.78)
Albumin (g/L) [median (range)]	39.90 (37.90–43.30)
Alanine aminotransferase (umol/s·L) [median (range)]	0.32 (0.25–0.59)
Aspartate aminotransferase (umol/s·L) [median (range)]	0.59 (0.45–0.79)
Alkaline phosphatase (umol/s·L) [median (range)]	2.60 (1.75–4.24)
Gamma-glutamyl transferase (umol/s·L) [median (range)]	2.04 (1.89–5.79)
Quick value (%) [median (range)]	91.50 (81.75–96.75)
INR [median (range)]	1.045 (1.020–1.115)
PTT (sec.) [median (range)]	27.30 (26.30–29.98)
Thrombin time (sec.) [median (range)]	16.65 (15.70–17.43)
C-reactive protein (mg/L) [median (range)]	8.75 (5.75–28.35)

**Table 2:** Complete blood counts one day before (pre T) and two days (post T) after selective internal radiotherapy radioembolization. Median value with 75% percentile is given. Nonparametric Wilcoxon matched-pairs signed rank test was performed and a *p* value below 0.05 was considered statistically significant.

Variables	Pre T	Post T	*p* Value
Leukocytes (Gpt/L) [median (range)]	7.34 (6.13–9.85)	9.62 (5.85–12.10)	0.2305
Erythrocytes (Tpt/L) [median (range)]	4.06 (3.76–4.40)	3.89 (3.61–4.31)	0.0001
red cell distribution width (%) [median (range)]	14.45 (13.18–15.33)	14.30 (13.45–15.85)	0.5723
Thrombocytes (Gpt/L) [median (range)]	217.00 (150.30–278.00)	201.00 (130.50–246.50)	<0.0001
Neutrophil granulocytes (%) [median (range)]	74.70 (66.55–80.00)	76.50 (66.93–81.75)	0.3750
Neutrophil granulocytes (Gpt/L) [median (range)]	5.42 (4.53–7.68)	6.58 (4.15–9.88)	0.7422
Immature granulocytes (%) [median (range)]	0.00 (0.00–0.01)	0.01 (0.00–0.23)	0.5000
Immature granulocytes (Gpt/L) [median (range)]	0.04 (0.02–0.06)	0.11 (0.04–0.14)	0.6875
Eosinophil granulocytes (%) [median (range)]	1.00 (0.53–2.00)	1.00 (0.00–1.80)	0.5000
Eosinophil granulocytes (Gpt/L) [median (range)]	0.07 (0.04–0.16)	0.04 (0.01–0.07)	0.5625
Basophil granulocytes (%) [median (range)]	0.65 (0.00–1.00)	0.30 (0.00–1.00)	0.7500
Basophil granulocytes (Gpt/L) [median (range)]	0.04 (0.02–0.06)	0.03 (0.02–0.04)	0.1250
Lymphocytes (%) [median (range)]	16.40 (10.25–20.30)	12.00 (9.00–19.80)	0.1563
Lymphocytes (Gpt/L) [median (range)]	1.19 (0.75–1.75)	1.18 (0.67–1.64)	0.1953
Monocytes (%) [median (range)]	9.00 (6.70–11.00)	8.00 (7.60–8.50)	0.8750
Monocytes (Gpt/L) [median (range)]	0.67 (0.43–0.92)	0.68 (0.45–1.00)	0.2500

**Table 3:** The correlation analyses between the parameters of the immune system assessed on extracellular vesicles (EVs) either with the administered activity dose (MBq) or with the interval until death (month) were performed by determining the Spearman correlation significance and Spearman r. Expression levels of cluster of differentiation (CD) proteins on the surface of EVs isolated from cholangiocellular carcinoma patients (CCA) one day before (pre T) and two days (post T) selective internal radiotherapy radioembolization were used. A *p* value below 0.05 was considered statistically significant. Abbreviations: HLA, human leukocyte antigen; MHC, major histocompatibility complex.

Parameter			Administered Activity Dose (MBq)		Interval until Death (Month)	
Immune System	EVs	Time Point	r	*p* Value	r	*p* Value
Lymphocytes	CD4	pre T	0.0446	0.8252	0.4290	0.1879
		post T	−0.0933	0.6956	0.1317	0.7168
	CD8	pre T	−0.1500	0.4125	0.0100	0.9719
		post T	−0.0498	0.8259	0.3174	0.3148
	CD44	pre T	−0.0357	0.8488	−0.0942	0.7384
		post T	0.2063	0.3571	−0.1360	0.6735
	CD69	pre T	0.0462	0.8051	−0.1122	0.6905
		post T	0.0271	0.9047	0.1284	0.6909
B cells	CD19	pre T	**0.3776**	**0.0331**	0.0978	0.7287
		post T	−0.1689	0.4523	0.0653	0.8487
	CD20	pre T	**0.3790**	**0.0355**	−0.2679	0.3158
		post T	0.0409	0.8602	0.1640	0.6105
NK cells	CD2	pre T	0.0205	0.9174	−0.2235	0.4424
		post T	−0.2465	0.2568	0.1705	0.6162
	CD8	pre T	−0.1500	0.4125	0.0100	0.9719
		post T	−0.0498	0.8259	0.3174	0.3148
	CD56	pre T	−0.1662	0.3148	0.2597	0.3699
		post T	−0.1617	0.4958	0.3016	0.3971
Platelets	CD41b	pre T	−0.2308	0.2117	0.4734	0.0873
		post T	0.0858	0.7192	0.0700	0.8381
	CD42a	pre T	−0.1941	0.9058	−0.0060	0.9825
		post T	−0.0518	0.8333	−0.0183	0.9600
	CD62P	pre T	−0.0993	0.6223	0.1600	0.5848
		post T	−0.2948	0.2070	−0.1213	0.7225
Leukocyte activation	CD45	pre T	−0.2652	0.1358	0.0849	0.7546
		post T	**−0.4881**	**0.0181**	0.1177	0.7157
	MHC II	pre T	**−0.4718**	**0.0064**	**0.5483**	**0.0227**
		post T	−0.3708	0.1075	−0.0340	0.9258
	CD14	pre T	0.2318	0.2263	0.0673	0.8270
		post T	−0.0627	0.7817	0.1498	0.6422
	CD29	pre T	**0.0181**	**0.0477**	0.1152	0.6950
		post T	−0.4287	0.0525	0.1446	0.6715
	CD40	pre T	**−0.3594**	**0.0400**	0.3061	0.2490
		post T	−0.2248	0.3145	0.1336	0.6952
	CD49e	pre T	−0.3289	0.0616	**0.5506**	**0.0271**
		post T	0.0628	0.7760	0.1819	0.5716
	HLA-ABC	pre T	−0.1789	0.3355	0.0377	0.8983
		post T	−0.1430	0.5591	−0.0322	0.9252
B cell activation	CD86	pre T	−0.0369	0.8463	0.5185	0.0575
		post T	0.2559	0.2504	0.0321	0.9211
Endothelial cells	CD31	pre T	−0.1417	0.1342	0.2048	0.5021
		post T	0.0448	0.8470	−0.2567	0.4205
	CD105	pre T	0.2721	0.1533	0.1248	0.6845
		post T	−0.0624	0.7937	−0.0886	0.7956
	CD146	pre T	0.1045	0.5759	0.0453	0.6845
		post T	−0.2027	0.3781	0.3811	0.2216
T and B cell adhesion markers	CD2	pre T	0.020	0.9174	−0.2235	0.4424
		post T	−0.2465	0.2568	0.1705	0.6162
	CD11c	pre T	−0.2713	0.1267	−0.0729	0.7884
		post T	**−0.4952**	**0.0163**	0.3815	0.2210
	CD29	pre T	**0.0181**	**0.0477**	0.1152	0.6950
		post T	−0.4287	0.0525	0.1446	0.6715
	CD31	pre T	−0.1417	0.5401	0.2048	0.5021
		post T	0.0448	0.8470	−0.2567	0.4205
	CD42a	pre T	−0.1941	0.2791	−0.0060	0.9825
		post T	−0.0518	0.8333	−0.0183	0.9600
	CD146	pre T	0.1045	0.5759	0.0453	0.8727
		post T	−0.2027	0.3781	0.3811	0.2216
	CD209	pre T	**0.4003**	**0.0232**	−0.4524	0.0785
		post T	−0.0554	0.8067	−0.0392	0.9037
Tissue Factor	CD142	pre T	**0.3579**	**0.0443**	−0.2336	0.3838
		post T	−0.0209	0.9265	0.2318	0.4685
“Don’t eat me signal”	CD24	pre T	−0.0095	0.9596	−0.2595	0.3502
		post T	−0.1191	0.5883	0.1287	0.7060
	CD44	pre T	−0.0357	0.8488	−0.0942	0.7384
		post T	0.2063	0.3571	−0.1360	0.6735
Stem cell/progenitor marker	CD133	pre T	0.1882	0.3106	0.2249	0.4204
		post T	0.0527	0.8206	0.3540	0.2854

## Data Availability

The data can be obtained upon a reasonable request from the corresponding author.
